# Alpha1-adrenoceptor antagonists block the positive chronotropic effect of 6-nitrodopamine in the isolated rat right atrium

**DOI:** 10.1590/1414-431X2026e15550

**Published:** 2026-10-12

**Authors:** B.L. Alves, A.J.S. Niederauer, J. Britto-Júnior, J.F. Sobanski, T.C.C. de Assis, D.L. Oliveira, G. Said, G.D. Mendes, A.S. Pupo, S. Marcondes, E. Antunes, G. De Nucci

**Affiliations:** 1 Departamento de Farmacologia, Faculdade de Ciências Médicas, Universidade Estadual de Campinas, Campinas, SP, Brasil; 2 Departamento de Farmacologia, Faculdade São Leopoldo Mandic, Campinas, SP, Brasil; 3 Departamento de Farmacologia, Faculdade de Medicina, Universidade Metropolitana de Santos, Santos, SP, Brasil; 4 Departamento de Biofísica e Farmacologia, Instituto de Biociências de Botucatu, Universidade Estadual Paulista, Botucatu, SP, Brasil; 5 Departamento de Farmacologia, Instituto de Ciências Biomédicas, Universidade de São Paulo, São Paulo, SP, Brasil

**Keywords:** Nitrocatecholamine, Isoprenaline, Cyclase-associated proteins, Adrenergic receptor

## Abstract

6-Nitrodopamine (6-ND) released from isolated rat atria produces potent positive chronotropic actions. Direct negative chronotropic responses to some selective α_1_-adrenoceptor (AR) antagonists like prazosin have been reported. Since α_1_-AR antagonists inhibit 6-ND-induced smooth muscle contractions, we have hypothesized that negative chronotropism induced by α_1_-AR antagonists reflects 6-ND blockade. This study investigated whether selective α_1_-AR antagonists affect 6-ND-induced positive chronotropism in isolated rat atria. Alfuzosin at 10 nM did not affect the atrial rate, but at 100 nM, it significantly reduced the atrial rate. This effect was not observed in L-NAME (100 mM)-treated preparations or in atria from rats chronically treated with L-NAME. Alfuzosin (100 nM) significantly reduced the 6-ND-induced positive chronotropism without affecting the chronotropic responses to noradrenaline, adrenaline, or dopamine. Similar results were obtained with doxazosin (1-10 nM), tamsulosin (10-100 nM), and prazosin (1-10 nM). Isoprenaline induced concentration-dependent increases in atrial rate, which were not significantly affected by any of these α_1_-AR antagonists. The results demonstrated that α_1_-AR antagonists reduced atrial basal rate only at concentrations affecting the positive chronotropic effect of 6-ND, and this reduction was not observed when NO synthesis was blocked, indicating that the negative chronotropic effect may involve 6-ND receptor blockade.

## Introduction

Endothelium-derived 6-nitrodopamine (6-ND) has been proposed as an important endogenous mediator of heart chronotropism and inotropism (1). Basal release of 6-ND has been detected in mouse, rat, and isolated rabbit atrium and ventricles by liquid chromatography coupled to tandem mass spectrometry (LC-MS/MS) (1). 6-ND added exogenously exerts a potent positive chronotropic effect in the isolated rat right atrium and synergizes with the classical catecholamines dopamine, noradrenaline, and adrenaline (2). Likewise, in isolated rat heart (Langendorff preparation), 6-ND significantly increases the left ventricular developed pressure (LVDP) and maximal rate of rise of left ventricular pressure parameters (dP/dtmax) (3). Release of 6-ND in atria and ventricles is significantly reduced in nitric oxide (NO)-deprived animals by using chronic treatment with the NO synthesis inhibitor Nω-nitro-L-arginine methyl ester (L-NAME) (3), confirming that synthesis of 6-ND is coupled to NO (3). Interestingly, the positive chronotropism induced by 6-ND in the isolated rat atrium is blocked by (±)-4-NO2-propranolol, indicating this compound behaves as a selective antagonist of 6-ND (4).

Apart from the cardiovascular system, 6-ND has been reported to be an important endogenous modulator of genitourinary smooth muscle contractility of animals and humans, including vas deferens (5), seminal vesicles (6), bladder (7), and corpus cavernosum (8). In the rat vas deferens, low concentrations (1 to 10 nM) of the α_1_-adrenoceptor (AR) antagonists prazosin, doxazosin, and tamsulosin markedly shifted the concentration-response curves to 6-ND to the right, without affecting the contractile responses to noradrenaline, adrenaline, or dopamine (9). In this concentration range, doxazosin and tamsulosin also significantly reduced the neurogenic vas deferens contractions induced by electric-field stimulation (EFS), indicating that 6-ND rather than noradrenaline is the major mediator of the ejaculatory process (10). In L-NAME-treated rats, these α_1_-AR antagonists at concentrations that blocked 6-ND induced contractions, failed to reduce the EFS-induced vas deferens contractions (10).

Although β-AR exhibits a major role in the regulation of heart function, the α_1_-AR, particularly the α_1A_ and α_1B_ subtypes, also produce a positive inotropic effect (11,12). Mouse right atrium expresses α_1A_-AR, α_1B_- AR, and α_1D_-AR mRNAs (13), but the exact role of α_1_-AR in regulating the isolated atrium preparation function remains poorly investigated. The positive inotropic and chronotropic actions induced by the selective α_1_-AR agonist phenylephrine in rats are partially decreased by the non-selective β-AR antagonist propranolol (14) and abolished when associated with the α_1_-AR antagonist prazosin (15). Non-selective α_1_-AR antagonists are known to exhibit the “first-dose phenomenon”, since oral administration causes a sudden and severe drop in blood pressure, especially during postural changes (16). Interestingly, prazosin does not cause reflex tachycardia; in fact, prazosin has been shown to produce a direct negative chronotropic effect (17).

Considering that 6-ND exerts a marked positive chronotropism in isolated atria and heart and that α_1_-AR blockers antagonize 6-ND responses in non-vascular smooth muscle tissue, we hypothesized that the negative chronotropic responses induced by the α1-AR antagonists are a consequence of 6-ND blockade in the heart. Therefore, in isolated rat atria preparation, we evaluated the changes in atrial rate in response to 6-ND, noradrenaline, adrenaline, dopamine, and isoprenaline in the absence and presence of different concentrations of the selective α_1_-AR antagonists tamsulosin (18), doxazosin (19), alfuzosin (20), and prazosin (21). The basal atrial rate was also evaluated in preparations pre-treated with either L-NAME or its inactive enantiomer D-NAME.

## Material and Methods

### Animals

Adult male Wistar rats (280-320 g) were obtained from two sources: the Central Animal House of the University of Campinas (CEMIB-UNICAMP; Brazil) and Animais de Laboratório Criação e Com. LTDA (ANILAB; Brazil). All experimental procedures were approved by the Ethics Committee for Animal Use of UNICAMP (CEUA; Protocol No. 6576-1/2024 and 6489-1/2024) in accordance with Brazilian Guidelines for the Production, Maintenance, and Use of Animals for Teaching or Research, as outlined by the National Council for the Control of Animal Experimentation (CONCEA) and the ARRIVE guidelines (22). The animals were housed in groups of three per cage, in ventilated cages with controlled conditions: humidity at 55±5%, temperature at 24±1°C, and a 12-h light-dark cycle. Animals had *ad libitum* access to filtered water and standard rodent chow (CR-1, Quimtia S.A., Brazil).

### Chronic L-NAME treatment

Rats were treated with the NO synthase inhibitor L-NAME, administered in drinking water at approximately 20 mg per rat per day (23). Control animals received only filtered water.

### Isolated rat right atrium preparation

Euthanasia was carried out by administering an overdose of isoflurane (greater than 5%) until one minute after breathing ceased. Exsanguination confirmed death. Following euthanasia, the heart was removed, and the right atrium was isolated and mounted between two metal hooks in custom-made 10-mL glass chambers filled with Krebs-Henseleit solution (KHS), continuously aerated with a 95% O_2_ and 5% CO_2_ mixture at 37°C, maintained by a heated circulator (PolyScience, USA). The tissues were equilibrated under a resting tension of 10 mN for one hour. The beating rate was assessed by directly measuring the mechanical force of contraction using a force transducer connected to the PowerLab data acquisition system (AD Instruments, Australia) and analyzed using LabChart Software, which captured the contractile activity of cardiac tissue in real-time. The atrial beating rate (in beats per minute: bpm) was calculated using the cyclic measurements tool in LabChart software, which detects cycles in the atrial force (mechanical force of contraction) to estimate the frequency of contractions. Throughout the experiments, the beating rate was continuously monitored to assess the physiological response to each drug. For acute effect analyses, the rate was measured at two main time points: immediately before drug administration (baseline) and 30 min after exposure. For concentration-response experiments, the atrial beating rate was recorded every 10 or 15 min, with a new drug concentration introduced at each interval.

### Effect of α_1_-AR antagonists on isolated rat right atria rate

The α1-AR antagonists alfuzosin, doxazosin, tamsulosin, and prazosin were systematically assessed as follows. Isolated rat right atria were equilibrated for 30 min, after which alfuzosin (100 nM), doxazosin (100 nM), tamsulosin (100 nM), or prazosin (10 nM) was added to the organ bath, and the atrial rate was monitored for an additional 30 min. The effects of these α1-AR antagonists were also assessed in atria pre-treated *in vitro* with L-NAME or its inactive enantiomer D-NAME (100 μM each for 30 min) (24), and in atria obtained from animals chronically treated with L-NAME (20 mg/rat/day). Time-matched control experiments were conducted using isolated atria without the addition of any drug to evaluate the natural variability in beating rate over time. The beating rate was measured at baseline and after 1 h.

In another series of experiments, the increase in atrial rate induced by 6-ND (1 pM, 30 min) was assessed after pre-treatment (30 min) with the antagonists alfuzosin (10 and 100 nM), doxazosin (1 and 10 nM), tamsulosin (10 and 100 nM), or prazosin (1 and 10 nM). Cumulative concentration-response curves for noradrenaline (1 nM-100 μM), adrenaline (1 nM-100 μM), dopamine (1 nM-1 mM), isoprenaline (1 pM-30 nM), and phenylephrine (1 nM-100 μM) were constructed both in the absence of antagonists and following a 30-min incubation with each antagonist (alfuzosin, doxazosin, tamsulosin, or prazosin, all at 100 nM or 1 μM). In all experiments, a separate atrium was used for each drug and concentration.

### Materials and drugs

Dopamine (H8502), isoprenaline (I0599990), Nω-nitro-L-arginine methyl ester (L-NAME; N5751), Nω-nitro-D-arginine methyl ester (D-NAME, N4770), and prazosin (P77910) were obtained from Sigma-Aldrich Chemical Co. (USA). Adrenaline (21245), alfuzosin (13648), and noradrenaline (35580) were purchased from Cayman Chemical Co. (USA). 6-Nitrodopamine (TRC N493720) was acquired from Toronto Research Chemicals (Canada). Tamsulosin (036934) was obtained from Swati Spentose Pvt. Ltd. (India). Doxazosin (N12345) was obtained from Nifty Labs Pvt. Ltd. (India). Sodium chloride (NaCl), potassium chloride (KCl), calcium chloride (CaCl_2_), magnesium sulfate (MgSO_4_), sodium bicarbonate (NaHCO_3_), potassium phosphate monobasic (KH_3_PO_4_), and glucose were acquired from Merck KGaA (Germany).

### Statistical analysis

Data are reported as means±SD. Comparisons between baseline values and those obtained during drug stimulation within the same sample were performed using a paired Student's *t*-test. Comparisons between two groups were conducted using an unpaired Student's *t*-test. Comparisons among three or more groups were evaluated using one-way analysis of variance (ANOVA), followed by Newman-Keuls test. A P-value <0.05 was considered statistically significant. Atrial rate data are reported either as the rate (beats per minute; bpm) before and after the respective stimulation or as the delta increase in atrial rate (bpm). The atrial rate is a calculated value, derived from various direct measurements of atrial activity in bpm.

## Results

### Effect of 6-ND on isolated rat right atria

The basal atrial rates before and after incubation with 6-ND (1 pM) and respective time-matched controls are shown in Figure 1. 6-ND (1 pM; Figure 1A) induced significant increases in atrial rate, reaching 14.0±4.0 bpm at 30 min and 20.0±6.0 bpm at 60 min (n=4) compared to time-matched controls (Figure 1B), which showed only 1.0±0.3 and 2.0±0.4 bpm at 30 and 60 min (n=4), respectively.

**Figure 1:**
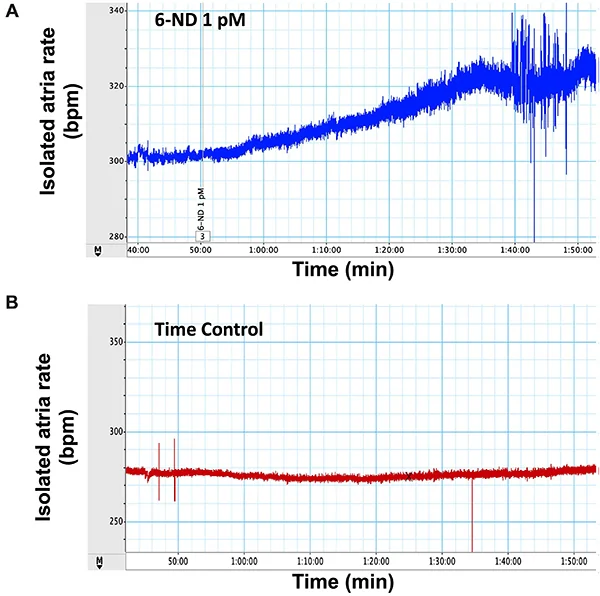
Representative traces showing the positive chronotropic effects of 6-nitrodopamine (6-ND, 1 pM) on isolated rat right atria at 60 min registration (**A**) in comparison with time-matched controls (**B**).

### Effect of the α_1_-selective AR antagonist alfuzosin on isolated rat right atria

Effects of 10 nM alfuzosin (30 min) did not alter the basal atrial rate (Figure 2A), but 100 nM caused a significant fall in atrial rate (Figure 2B). Representative atrial-rate traces in the presence of the α1-selective AR antagonist alfuzosin (100 nM) are shown in Supplementary Figure S1A. Pre-incubation (30 min) of the atria with 6-ND (1 pM) alone caused significant increases in atrial rate (Figure 2C), which were unaffected when the atria were incubated with alfuzosin at 10 nM but significantly reduced when incubated with alfuzosin at 100 nM (Figure 2C). Alfuzosin at 1 µM significantly blocked 6-ND (1 pM)-induced increases of atrial rate (277±10 and 285±10 bpm in absence and presence of alfuzosin, respectively; P=0.5670; n=4). Table 1 shows means±SD for the 6-ND (1 pM) responses in the absence and presence of alfuzosin.

**Figure 2:**
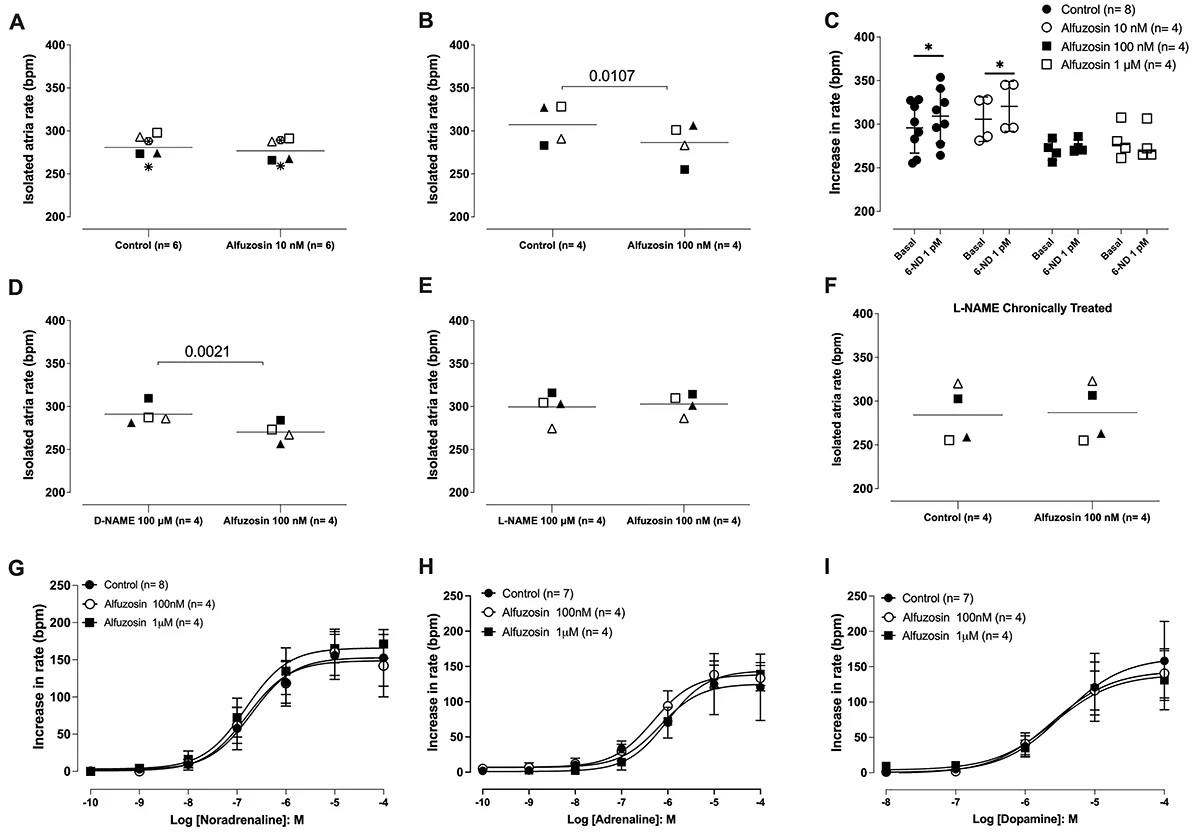
Antagonism of the positive chronotropic responses of 6-nitrodopamine (6-ND) by the a_1_-adrenergic receptor antagonist alfuzosin. Alfuzosin at 10 nM (30 min (**A**) and at 100 nM (**B**). At 100 nM and 1 μM, alfuzosin effectively blocked the increase in atrial rate induced by 6-ND (**C**). In D-NAME (100 μM, 30 min)-treated preparations, alfuzosin (100 nM, 30 min) significantly reduced the atrial rate (**D**), an effect not observed with L-NAME *in vitro* (100 μM, 30 min; **E**) or in atria from animals chronically treated with L-NAME (**F**). Effect of alfuzosin (100 nM and 1 μM; 30 min) on concentration-response curves induced by noradrenaline (**G**), adrenaline (**H**), and dopamine (**I**). Data are reported as means±SD. *P<0.05. **A**, **B**, **D**, **E**, and **F** show individual atrial responses, where each symbol represents a single atrium. Student's *t*-test and ANOVA.

**Table 1:** Effect of selective α_1_-adrenoceptor antagonists on the positive chronotropic effect of 6-nitrodopamine (6-ND) in isolated rat right atria.

Drug	BPM	P-value	(n)
Alfuzosin			
Control	297±29		8
6-ND 1 pM	308±31	0.0006	8
Alfuzosin 10 nM	307±26		4
Alfuzosin 10 nM + 6-ND 1 pM	320±32	0.0302	4
Alfuzosin 100 nM	270±12		4
Alfuzosin 100 nM + 6-ND 1 pM	274±8	0.2642	4
Alfuzosin 1 µM	280±20		4
Alfuzosin 1 μM+ 6-ND 1 pM	277±20	0.4899	4
Doxazosin			
Control	290±7		9
6-ND 1 pM	301±8	0.0278	9
Doxazosin 1 nM	289±8		6
Doxazosin 1 nM + 6-ND 1 pM	302±7	0.0299	6
Doxazosin 10 nM	284±15		4
Doxazosin 10 nM + 6-ND 1 pM	286±13	0.9203	4
Doxazosin 100 nM	266±16		4
Doxazosin 100 nM + 6-ND 1 pM	269±21	0.9203	4
Tamsulosin			
Control	306±18		8
6-ND 1 pM	317±20	0.0003	8
Tamsulosin 10 nM	304±22		4
Tamsulosin 10 nM + 6-ND 1 pM	316±22	0.0218	4
Tamsulosin 100 nM	265±16		4
Tamsulosin 100 nM + 6-ND 1 pM	266±17	0.2221	4
Tamsulosin 1 μM	271±20		4
Tamsulosin 1 μM+ 6-ND 1 pM	273±20	0.8469	4
Prazosin			
Control	307±11		9
6-ND 1 pM	325±11	0.0031	9
Prazosin 1 nM	290±20		6
Prazosin 1 nM + 6-ND 1 pM	306±26	0.0083	6
Prazosin 10 nM	291±21		9
Prazosin 10 nM + 6-ND 1 pM	299±24	0.0208	9
Prazosin 100 nM	269±19		4
Prazosin 100 nM + 6-ND 1 pM	263±27	0.7340	4

Data are reported as means and SD. BPM: beats per minute.

In D-NAME (100 µM)-treated preparations, alfuzosin significantly reduced the atrial rate (Figure 2D), whereas in L-NAME (100 µM)-treated preparations alfuzosin (100 nM) had no effect on the atrial basal rate (Figure 2E). In atria obtained from animals chronically treated with L-NAME, incubation with alfuzosin (100 nM) did not affect the basal atrial rate (Figure 2F). Table 2 shows means±SD for the chronotropic responses in the absence and presence of alfuzosin in preparations treated with D-NAME or L-NAME.

**Table 2:** Basal atrial rates in response to the α_1_-adrenoceptor antagonists alfuzosin, doxazosin, tamsulosin, and prazosin in isolated rat right atria in the absence and presence of D-NAME or L-NAME.

Drug/Treatments	BPM/SD	P-value	(n)
Alfuzosin			
Control	281±15		6
Alfuzosin 10 nM	277±15	0.6483	6
Control	307±24		4
Alfuzosin 100 nM	286±22	0.0107	4
D-NAME	291±12		4
D-NAME + Alfuzosin 100 nM	270±14	0.0021	4
L-NAME	299±16		4
L-NAME + Alfuzosin 100 nM	302±12	0.1906	4
Chronically treated L-NAME (Control)	284±32		4
Chronically treated L-NAME + Alfuzosin 100 nM	286±32	0.4554	4
Doxazosin			
Control	300±12		6
Doxazosin 1 nM	301±15	0.9790	6
Control	316±32		4
Doxazosin 10 nM	304±26	0.0109	4
D-NAME	284±16		4
D-NAME + Doxazosin 100 nM	256±24	0.090	4
L-NAME	247±12		4
L-NAME + Doxazosin 100 nM	243±12	0.1783	4
Chronically treated L-NAME (Control)	255±20		4
Chronically treated L-NAME + Alfuzosin 100 nM	259±20	0.4008	4
Tamsulosin			
Control	245±20		4
Tamsulosin 10 nM	240±22	0.5786	4
Control	295±18		4
Tamsulosin 100 nM	271±26	0.0488	4
D-NAME	312±29		5
D-NAME + Tamsulosin 100 nM	268±16	0.0246	5
L-NAME	316±12		6
L-NAME + Tamsulosin 100 nM	312±10	0.1501	6
Chronically treated L-NAME (Control)	262±26		4
Chronically treated L-NAME + Tamsulosin 10 nM	264±28	0.4558	4
Prazosin			
Control	295±22		4
Prazosin 1 nM	294±22	0.4824	4
Control	316±8		4
Prazosin 10 nM	301±6	0.0239	4
D-NAME	322±38		5
D-NAME + Prazosin 10 nM	305±44	0.0314	5
L-NAME	295±19		6
L-NAME + Prazosin 10 nM	297±15	0.4200	6
Chronically treated L-NAME (Control)	264±20		4
Chronically treated L-NAME + Prazosin 10 nM	265±24	0.4895	4

BPM/SD: beats per minute±standard deviation.

Incubation with alfuzosin (100 nM and 1 µM) had no effect on the positive chronotropic effects induced by noradrenaline (Figure 2G), adrenaline (Figure 2H), and dopamine (Figure 2I). Table 3 shows means±SD for pEC_50_ (potency) and Emax (maximum effect) of the positive chronotropic responses induced by noradrenaline, adrenaline, and dopamine in the absence and presence of alfuzosin.

**Table 3:** Effect of selective α_1_-adrenoceptor antagonists on pEC_50_ and Emax of noradrenaline-, adrenaline-, dopamine-, phenylephrine-, and isoprenaline-induced positive chronotropic responses in isolated rat right atria.

Drug	pEC_50_	P-value	Emax	P-value	(n)
Alfuzosin					
Noradrenaline					
Control	6.69±0.28		153±38		8
Alfuzosin (100 nM)	6.75±0.13	0.6733	142±42	0.6734	4
Alfuzosin (1 μM)	6.74±0.45	0.9556	171±19	0.2569	4
Adrenaline					
Control	6.20±0.46		120±46		7
Alfuzosin (100 nM)	6.32±0.19	0.6217	133±18	0.6157	4
Alfuzosin (1 μM)	6.02±0.11	0.4965	139±16	0.4688	4
Dopamine					
Control	5.37±0.37		158±56		7
Alfuzosin (100 nM)	5.61±0.09	0.2624	140±34	0.5870	4
Alfuzosin (1 μM)	5.68±0.35	0.2136	130±41	0.4207	4
Phenylephrine					
Control	5.27±0.46		141±12		4
Alfuzosin (100 nM)	5.00±0.22	0.1609	158±7	0.0320	4
Alfuzosin (1 μM)	4.43±0.13	0.0382	130±4	0.4608	4
Isoprenaline					
Control	9.90±0.08		131±19		4
Alfuzosin (100 nM)	9.48±0.48	0.1397	134±22	0.7617	4
Doxazosin					
Noradrenaline					
Control	6.08±0.23		166±30		8
Doxazosin (100 nM)	5.93±0.79	0.5976	176±59	0.7246	4
Doxazosin (1 μM)	5.90±0.37	0.9542	173±22	0.9279	4
Adrenaline					
Control	6.36±0.70		163±17		8
Doxazosin (100 nM)	6.40±0.39	0.9233	168±19	0.7015	4
Doxazosin (1 μM)	6.09±0.30	0.2369	172±12	0.7214	4
Dopamine					
Control	4.86±0.39		171±47		8
Doxazosin (100 nM)	4.90±0.27	0.8586	198±11	0.2852	4
Doxazosin (1 μM)	4.93±0.61	0.9426	181±13	0.1185	4
Phenylephrine					
Control	5.31±0.15		142±12		4
Doxazosin (100 nM)	5.18±0.13	0.1483	154±14		4
Doxazosin (1 μM)	4.96±0.33	0.0790	133±38	0.2098	4
Isoprenaline					
Control	9.60±0.91		127±59		4
Doxazosin (100 nM)	10.29±0.19	0.1902	108±25	0.7451	4
Tamsulosin					
Noradrenaline					
Control	6.67±0.43		158±22		8
Tamsulosin (100 nM)	6.98±0.19	0.2012	142±36	0.3386	4
Tamsulosin (1 μM)	6.75±0.36	0.2987	150±38	0.7388	6
Adrenaline					
Control	6.07±0.35		120±47		7
Tamsulosin (100 nM)	6.28±0.21	0.3727	133±18	0.6157	4
Tamsulosin (1 μM)	6.00±0.12	0.1145	139±16	0.6508	4
Dopamine					
Control	5.42±0.21		204±29		8
Tamsulosin (100 nM)	5.44±0.39	0.8754	202±34	0.9029	4
Tamsulosin (1 μM)	5.26±0.26	0.4649	186±29	0.4563	4
Phenylephrine					
Control	5.51±0.13		156±14		4
Tamsulosin (100 nM)	5.45±0.16		154±15	0.4377	4
Tamsulosin (1 μM)	5.22±0.18	0.2714	140±14	0.0860	4
Isoprenaline					
Control	9.83±0.29	0.0241	159±23		4
Tamsulosin (100 nM)	9.55±0.09	0.1141	133±21	0.1461	4
Prazosin					
Noradrenaline					
Control	6.38±0.48		188±29		8
Prazosin (100 nM)	6.47±0.26	0.4378	190±11	0.9061	4
Prazosin (1 μM)	5.97±0.14	0.0158	172±21	0.1666	4
Adrenaline					
Control	6.42±0.38		175±46		8
Prazosin (100 nM)	6.48±0.34	0.8115	153±64	0.5284	4
Prazosin (1 μM)	6.78±0.37	0.2668	158±69	0.9195	4
Dopamine					
Control	5.42±0.43		171±43		8
Prazosin (100 nM)	5.14±0.27	0.2775	177±56	0.8546	4
Prazosin (1 μM)	5.48±0.18	0.0819	177±23	0.9778	4
Phenylephrine					
Control	5.42±0.18		136±32		4
Prazosin (100 nM)	5.35±0.28	0.1773	124±7	0.2488	4
Prazosin (1 μM)	5.09±0.60	0.0948	108±30	0.0603	4
Isoprenaline					
Control	10.12±0.27		130±50		4
Prazosin (100 nM)	9.94±0.26	0.3650	113±49	0.6562	4

pEC_50_: potency (mean±SD); Emax: maximum effect (mean±SD bpm). ANOVA.

### Effect of the α_1_-selective AR antagonist doxazosin on isolated rat right atria

Doxazosin at 1 nM (30 min) did not alter the basal atrial rate (Figure 3A), but at 10 nM it caused a significant fall in atrial rate (Figure 3B). Representative atrial-rate traces in the presence of the α1-selective AR antagonist doxazosin (10 nM) are shown in Supplementary Figure S1B. Incubation (30 min) of the atria with 6-ND (1 pM) caused a significant increase in atrial rate (Figure 3C), which was not affected when the atria were incubated with doxazosin at 1 nM but significantly reduced when incubated with doxazosin at 10 nM (Figure 3C). Doxazosin at 100 nM significantly blocked 6-ND (1 pM)-induced increases of atrial rate (301±14 and 308±11 bpm in the absence and presence of doxazosin, respectively; P=0.6692; n=4). Table 1 shows means±SD for the 6-ND (1 pM) responses in the absence and presence of doxazosin.

**Figure 3:**
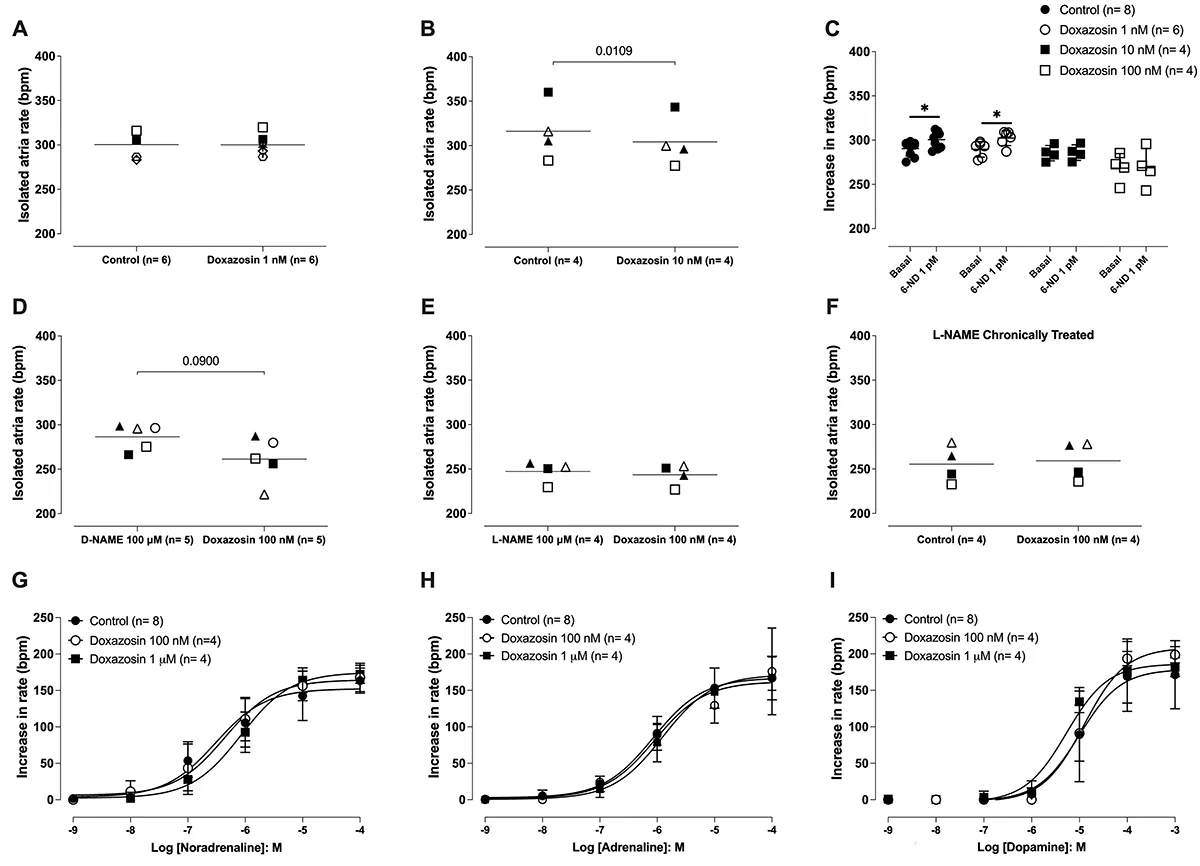
Antagonism of the positive chronotropic responses of 6-nitrodopamine (6-ND) by the α1-adrenoceptor antagonist doxazosin. Doxazosin at 1 nM (30 min; **A**) did not affect basal atrial rate, but at 10 nM (**B**) caused a significant reduction. At 10 and 100 nM, doxazosin (30 min) effectively blocked the increase in atrial rate induced by 6-ND (**C**). In D-NAME (100 μM, 30 min)-treated preparations, doxazosin (100 nM, 30 min) significantly reduced the atrial rate (**D**), an effect not observed with L-NAME *in vitro* (100 μM, 30 min; **E**) or in atria from animals chronically treated with L-NAME (**F**). Doxazosin (100 nM and 1 μM; 30 min) had no effect on concentration-response curves induced by noradrenaline (**G**), adrenaline (**H**), and dopamine (**I**). Data are reported as means±SD. *P<0.05. Student's *t*-test and ANOVA. **A**, **B**, **D**, **E**, and **F** show individual atrial responses, where each symbol represents a single atrium.

In atria pre-treated (30 min) with D-NAME (100 µM), doxazosin (100 nM) induced a significant fall in atrial basal rate (Figure 3D), whereas in the presence of L-NAME doxazosin had no effect on the atrial rate (100 µM; Figure 3E). In atria obtained from animals chronically treated with L-NAME, incubation with doxazosin (100 nM) also failed to affect the basal atrial rate (Figure 3F). Table 2 shows means±SD for the chronotropic responses in the absence and presence of doxazosin in preparations treated with D-NAME or L-NAME.

Incubation with doxazosin (100 nM and 1 µM) had no effect on the positive chronotropic effects induced by noradrenaline (Figure 3G), adrenaline (Figure 3H), and dopamine (Figure 3I). Table 3 shows means±SD for pEC_50_ and Emax of the positive chronotropic responses induced by noradrenaline, adrenaline, and dopamine in the absence and presence of doxazosin.

### Effect of the α_1_-selective AR antagonist tamsulosin on isolated rat right atria

Tamsulosin at 10 nM (30 min) did not alter the basal atrial rate (Figure 4A), but at 100 nM it caused a significant fall in atrial rate (Figure 4B). Representative atrial rate traces in the presence of the α_1_-selective AR antagonist tamsulosin (100 nM) are shown in Supplementary Figure S1C. Incubation of the atria with 6-ND (1 pM) for 30 min caused a significant increase in atrial rate (Figure 4C). This increase was not affected when the atria were incubated with tamsulosin at 10 nM, but significantly reduced when incubated with tamsulosin at 100 nM (Figure 4C). Tamsulosin at 1 µM significantly blocked the 6-ND (1 pM)-induced increases of atrial rate (306±3 bpm in the absence and 311±28 bpm in the presence of tamsulosin; P=0.7892; n=4). Table 1 shows means±SD for the 6-ND (1 pM) responses in the absence and presence of tamsulosin.

**Figure 4:**
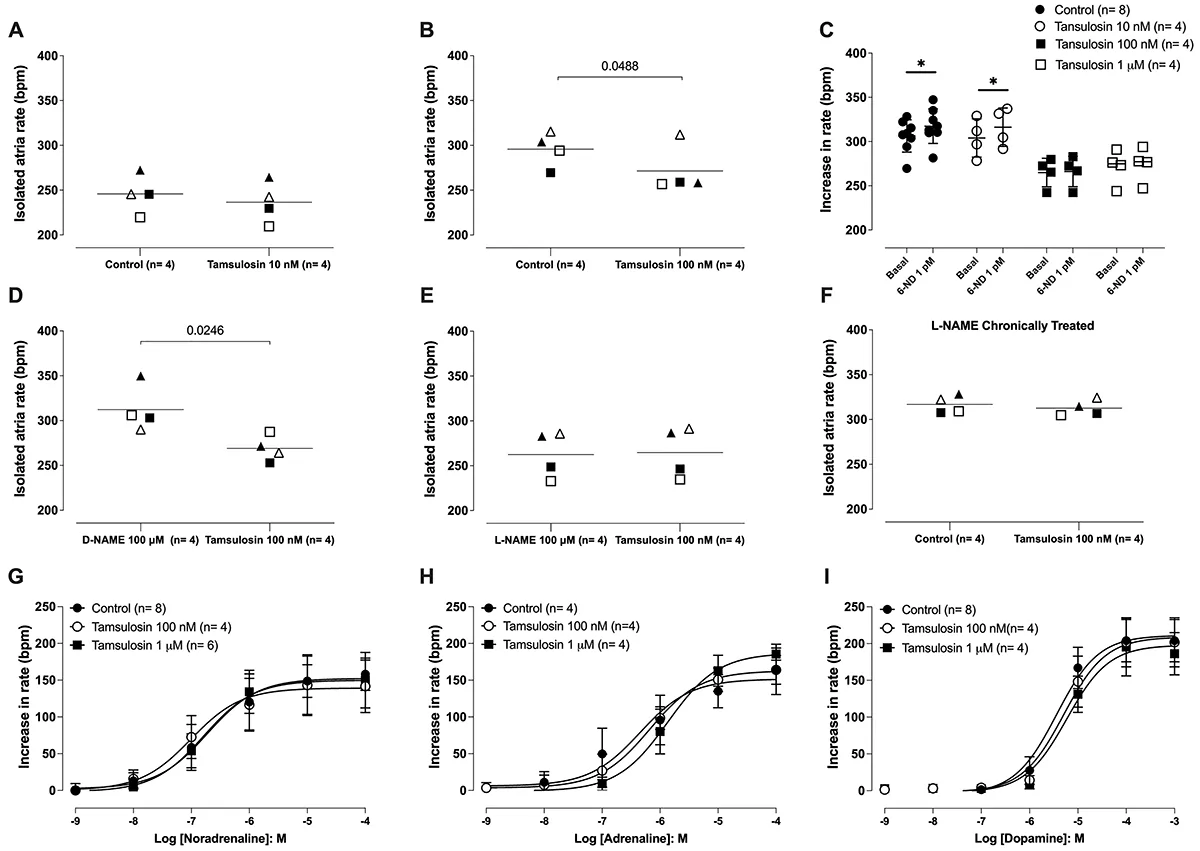
Antagonism of the positive chronotropic responses of 6-nitrodopamine (6-ND) by the α1-adrenergic receptor antagonist tamsulosin. Tamsulosin at 10 nM (30 min; **A**) did not affect the basal atrial rate, but at 100 nM (**B**) caused a significant reduction of the atrial rate. At 100 nM and 1 μM, tamsulosin (30 min) effectively blocked the increase in atrial rate induced by 6-ND (**C**). In D-NAME (100 μM, 30 min)-treated preparations, tamsulosin (100 nM, 30 min) significantly reduced the atrial rate (**D**), an effect not observed with L-NAME *in vitro* (100 μM, 30 min; **E**) or in atria obtained from animals chronically treated with L-NAME (**F**). Tamsulosin (100 nM and 1 μM; 30 min) had no effect on the concentration-response curves induced by noradrenaline (**G**), adrenaline (**H**), and dopamine (**I**). Data are reported as means±SD. *P<0.05. Student's *t*-test and ANOVA. **A**, **B**, **D**, **E**, and **F** show individual atrial responses, where each symbol represents a single atrium.

In atria pre-treated (30 min) with D-NAME (100 µM), tamsulosin (100 nM) induced a significant drop in atrial basal rate (Figure 4D); however, in the presence of L-NAME, tamsulosin (100 µM) had no effect on the atrial rate (Figure 4E). In atria from animals chronically treated with L-NAME, incubation with tamsulosin (100 nM) had no effect on the basal atrial rate (Figure 4F). Table 2 shows means±SD for the chronotropic responses in the absence and presence of tamsulosin in preparations treated with D-NAME or L-NAME.

Incubation with tamsulosin (100 nM and 1 µM) had no effect on the positive chronotropic effects induced by noradrenaline (Figure 4G), adrenaline (Figure 4H), and dopamine (Figure 4I). Table 3 shows means±SD for pEC_50_ and Emax of the positive chronotropic responses induced by noradrenaline, adrenaline, and dopamine in the absence and presence of tamsulosin.

### Effect of the α_1_-selective AR antagonist prazosin on isolated rat right atria

Prazosin at 1 nM (30 min) did not alter the basal atrial rate (Figure 5A), but at 10 nM caused a significant fall in atrial rate (Figure 5B). Representative atrial-rate traces in the presence of the α1-selective AR antagonist prazosin (10 nM) are shown in Supplementary Figure S1D. Incubation of the atria with 6-ND (1 pM) for 30 min caused a significant increase in atrial rate (Figure 5C), and this increase was not affected when the atria were incubated with prazosin at 1 nM or 10 nM but significantly reduced when incubated with prazosin at 100 nM (Figure 5C). Prazosin at 100 nM significantly blocked 6-ND-(1 pM)-induced increases of atrial rate (288±14 bpm in the absence and 289±11 bpm in the presence of prazosin; P=0.96482; n=4). Table 1 shows means±SD for the 6-ND (1 pM) responses in the absence and presence of prazosin.

**Figure 5:**
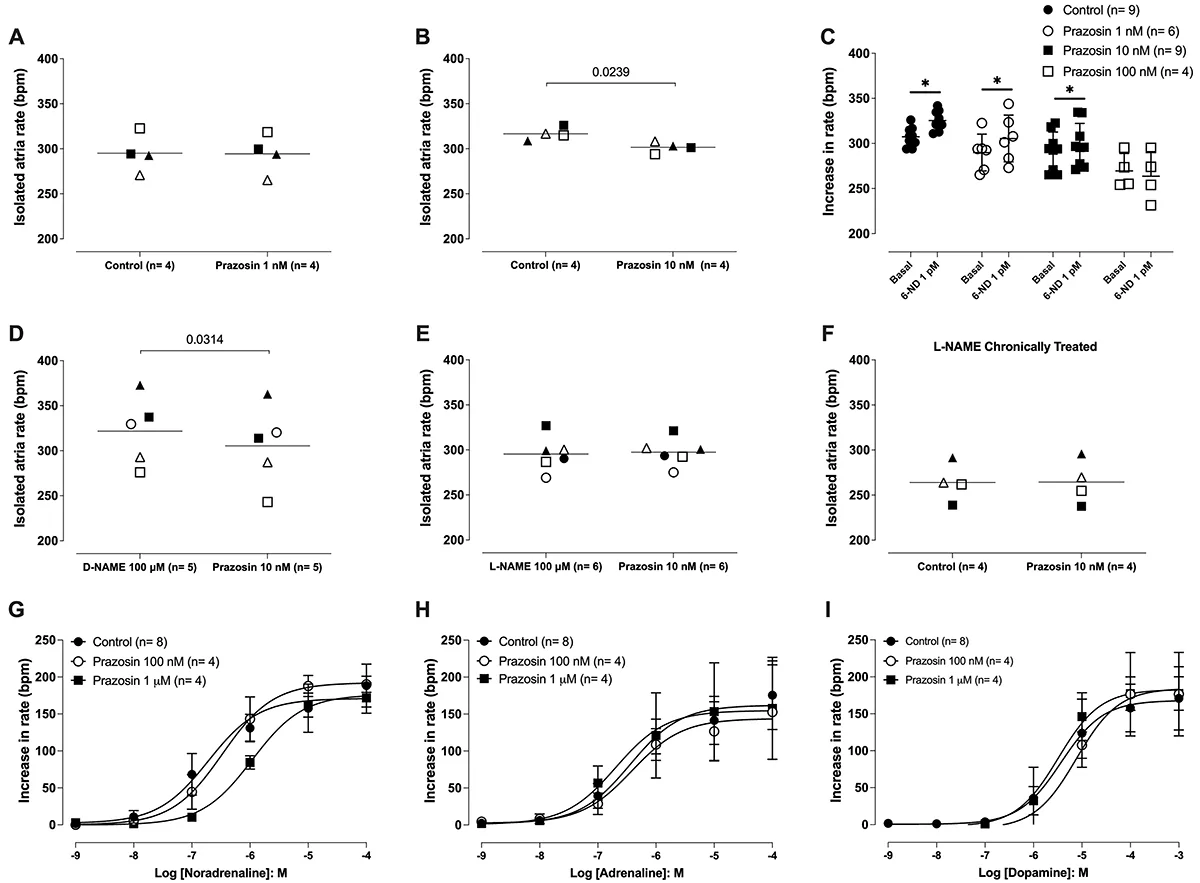
Antagonism of the positive chronotropic responses of 6-nitrodopamine (6-ND) by the α1-adrenoceptor antagonist prazosin. Prazosin at 1 nM (30 min; **A**) did not affect the basal atrial rate, but at 10 nM (**B**) caused a significant reduction of the atrial rate. At 100 nM, prazosin (30 min) effectively blocked the increase in atrial rate induced by 6-ND (**C**). In D-NAME (100 μM, 30 min)-treated preparations, prazosin (10 nM, 30 min) significantly reduced the atrial rate (**D**), an effect neither observed with L-NAME *in vitro* (100 μM, 30 min; **E**) nor in atria obtained from animals chronically treated with L-NAME (**F**). Prazosin (100 nM and 1 μM; 30 min) had no effect on the concentration-response curves induced by noradrenaline (**G**), adrenaline (**H**), and dopamine (**I**). Data are reported as means±SD. *P<0.05. Student's *t*-test and ANOVA. **A**, **B**, **D**, **E**, and **F** show individual atrial responses, where each symbol represents a single atrium.

In atria pre-treated (30 min) with D-NAME (100 µM), prazosin (10 nM) induced a significant fall in atrial basal rate (Figure 5D); however, in the presence of L-NAME (100 µM), prazosin had no effect on the atrial rate (Figure 5E). In atria from animals chronically treated with L-NAME, incubation with prazosin (10 nM) had no effect on the basal atrial rate (Figure 5F). Table 2 shows means±SD for the chronotropic responses in the absence and presence of prazosin in preparations treated with D-NAME or L-NAME.

Incubation with prazosin (100 nM or 1 µM) had no effect on the positive chronotropic effects induced by adrenaline (Figure 5H) and dopamine (Figure 5I). However, prazosin at 1 µM caused a rightward shift in the noradrenaline concentration-response curve (Figure 5G). Table 3 shows means±SD for pEC_50_ and Emax of the positive chronotropic responses induced by noradrenaline, adrenaline, and dopamine in the absence and presence of prazosin.

### Effect of α_1_-selective AR antagonists on the chronotropic effect induced by phenylephrine

Phenylephrine induced concentration-dependent increases in the atrial rate, and these increases were not affected by pre-treatment with alfuzosin (100 nM; Figure 6A), doxazosin (100 nM; Figure 6B), tamsulosin (100 nM; Figure 6C), and prazosin (100 nM; Figure 6D). However, the higher concentration (1 μM) of each antagonist caused a rightward shift of the concentration-response curves. Table 3 shows means±SD for pEC_50_ and Emax of the positive chronotropic responses induced by phenylephrine in the absence and presence of α1-AR antagonists.

**Figure 6:**
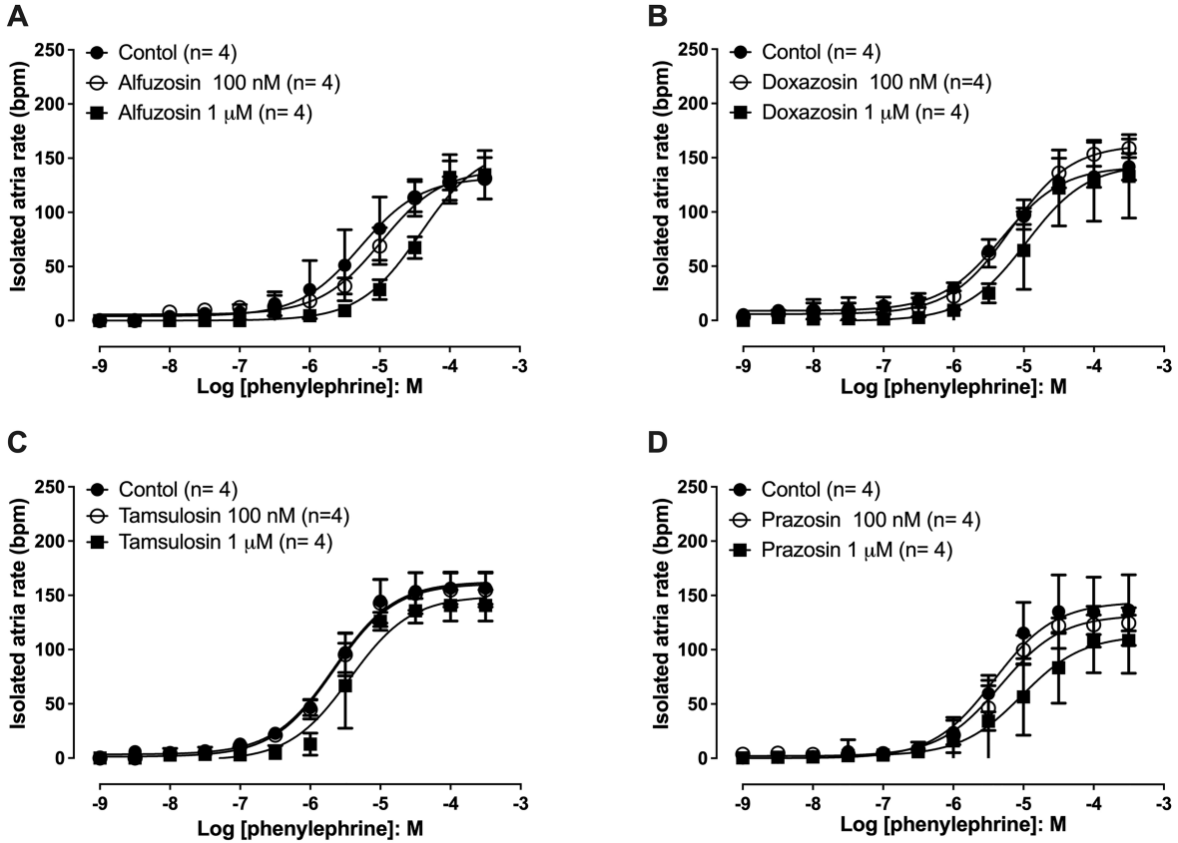
Effect of selective α_1_-adrenoceptor antagonists on the positive chronotropic effects induced by phenylephrine. Isolated atria were pre-treated for 30 min with alfuzosin (100 nM or 1 μM; **A**), doxazosin (100 nM or 1 μM; **B**), tamsulosin (100 nM or 1 μM; **C**), or prazosin (100 nM or 1 μM; **D**) before performing cumulative concentration-response curves to phenylephrine. Data are reported as means±SD (n=4). ANOVA.

### Effect of α_1_-selective AR antagonists on the chronotropic effect induced by isoprenaline

Isoprenaline induced concentration-dependent increases in the atrial rate, and these increases were not affected by pre-treatments with alfuzosin (100 nM; Figure 7A), doxazosin (100 nM; Figure 7B), tamsulosin (100 nM; Figure 7C), and prazosin (100 nM; Figure 7D). Table 3 shows means±SD for pEC_50_ and Emax of the positive chronotropic responses induced by isoprenaline in the absence and presence of α1-AR antagonists.

**Figure 7:**
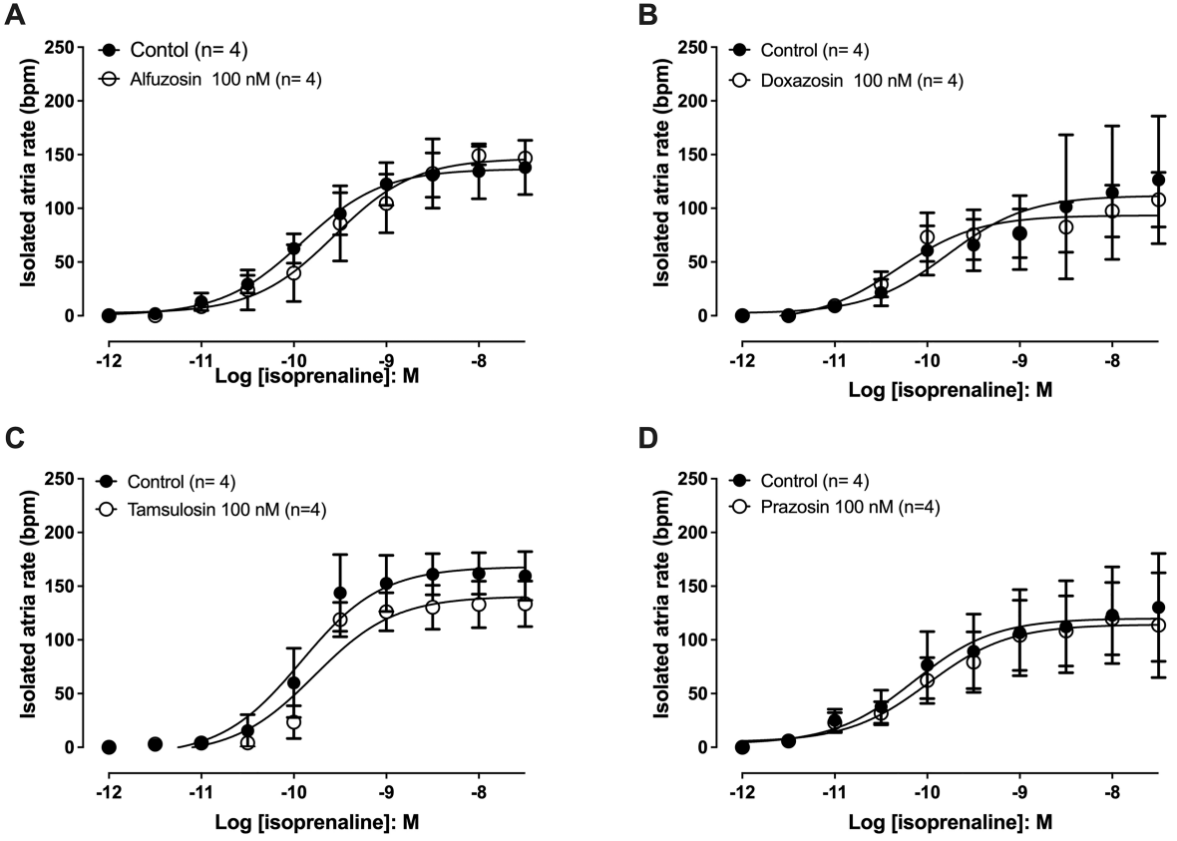
Lack of effect of selective α_1_-adrenoceptor antagonists on the positive chronotropic effects induced by isoprenaline. Isolated atria were pre-treated for 30 min with alfuzosin (100 nM; **A**), doxazosin (100 nM; **B**), tamsulosin (100 nM; **C**), or prazosin (100 nM; **D**). The control was not treated. Cumulative concentration-response curves to isoprenaline were then obtained in response to these pre-treatments. Data are reported as means±SD (n=4). ANOVA.

## Discussion

### Main new findings

The role of α_1_-AR on cardiac chronotropism is controversial and may depend on the animal species investigated and the experimental design. For instance, in spontaneously beating right atria of rats or cats, a positive chronotropic effect mediated by α-AR has not been demonstrated (25). However, in the pithed rat, the “selective” α_1_-AR agonist amidephrine caused a positive chronotropic response, which was antagonized by prazosin but not by propranolol (26), indicating that α_1_-AR can mediate chronotropic responses in the rat *in vivo*.

The positive chronotropic effect induced by the α_1_-AR agonist phenylephrine was rightward shifted by prazosin in preparations pre-treated with propranolol, suggesting that the α_1_-AR component in the atria can only be unmasked in the presence of β_1_/β_2_-AR blockade (27). Moreover, the positive chronotropic effect induced by the α_1_-AR agonist methoxamine in isolated rat atria was unaffected by pre-treatment with propranolol but was abolished by pre-treatment with the non-selective α_1_-AR antagonist phentolamine (28).

Our results clearly demonstrated that the α_1_-AR antagonists alfuzosin, doxazosin, tamsulosin, and prazosin provoked significant reductions in the atrial basal rate only at concentrations that affect the positive chronotropic effect of 6-ND. Although β-AR are considered to play a major role in the mammalian heart, it is unlikely that the α_1_-AR antagonists act on β-AR. For instance, in Chinese hamster ovary (CHO) epithelial cell lines expressing either human β_1_-AR (29) or human β_2_-AR (30), alfuzosin presented no binding to β_1_-AR, and the log KD for β_2_-AR was -4.18 (approximately 70 μM) (31), a concentration seven hundred times higher than that required for alfuzosin to reduce atrial basal rate and to inhibit 6-ND-induced positive chronotropic effect (100 nM). Doxazosin log KD for β_1_- and β_2_-AR are approximately 20 and 3 μM, respectively, but it reduced atrial rate and inhibited the 6-ND chronotropic effect at 10 nM. Like alfuzosin, prazosin presented no binding to β_1_-AR, and the log KD for β_2_-AR was approximately 8 μM, but it reduced both the atrial rate and 6-ND-induced chronotropism at 10 nM. In addition, at concentrations that the α_1_-AR antagonists caused significant reductions in atrial basal rate, they did not affect the positive chronotropic effects induced by dopamine, noradrenaline, or adrenaline. Moreover, at concentrations that reduced basal atrial rate, they did not affect the increase in heart rate induced by either the α_1_-AR agonist phenylephrine (32,33) or the β_1_/β_2_-AR agonist isoprenaline (33).

As observed with the β_1_-AR antagonists atenolol, betaxolol, and metoprolol (1), the fall in atrial basal rate induced by the α_1_-AR antagonists was not observed if the atria were pre-treated with the NO synthase inhibitor L-NAME or if the atria were from animals chronically treated with L-NAME, indicating that the mechanism of action responsible for the negative chronotropic effect could be the blockade of the 6-ND receptor, as proposed for the β-blockers (1). This explanation is supported by lack of effect of the inactive enantiomer D-NAME (24) on the α_1_-AR antagonist-induced negative chronotropic effect. Another possible explanation is that the α1-AR antagonists act as inverse agonists, as shown in α_1_a- and α_1_b-adrenoceptor subtypes (34). For instance, the β-adrenoceptor antagonists nebivolol and bisoprolol exhibit inverse agonist activity in human and atrial myocardium (35), although this finding is controversial (36) and may be restricted to mutant β_1_-adrenoceptors (37). However, this is unlikely, since inverse agonism does not explain the lack of effect of the α1-adrenoceptor antagonists in L-NAME-treated atria or in atria obtained from L-NAME-treated rats.

Cyclase-associated proteins 1 (CAP-1) and CAP-2 have been identified as potential receptors for 6-ND in human induced pluripotent stem cell (hiPSC)-derived cardiomyocytes (38). Reduction of atrial rate by β-adrenoceptor antagonists is associated with blockade of the 6-ND receptor (1), and depletion of CAP1 in SW480 and HCT116 colon cancer cells abolished the stimulatory effects of the β-AR agonist isoproterenol (39). Binding studies with labeled 6-ND should provide further information on the interactions between both α1- and β1-adrenoceptor antagonists with the 6-ND receptor in the isolated rat right atrium. Nevertheless, the finding that the fall in atrial rate induced by α1-AR antagonists occurs only at concentrations that blocked the positive chronotropic effect induced by 6-ND reinforces the concept of this novel catecholamine as a major mediator of heart chronotropism.

### Clinical relevance

The identification that alpha1-blockers block the 6-ND receptor in atria may explain the paradoxical negative chronotropic effect observed in patients.

### Limitations of the study

This was an *in vitro* study and although rat and human hearts share many morphological and innervation similarities, significant differences exist, primarily due to the much faster heart rate in rats, which requires different calcium handling and molecular characteristics compared to the human heart. Only atria from male rats were employed; therefore, caution is needed when extrapolating the data to female rats. However, α1-AR antagonists are commonly used for lower urinary tract symptoms, and no dose adjustment based on patient sex is required (40).

## Data Availability

The data that support the findings of this study are available from the corresponding author upon reasonable request.

## Supplementary Materials

Click to view [pdf].
