# Supplementary material for: Alpha1-adrenoceptor antagonists block the positive chronotropic effect of 6-nitrodopamine in the isolated rat right atrium
Source: Braz J Med Biol Res. 2026 Oct 12;59:e15550. doi: 10.1590/1414-431X2026e15550 (PMC13660870; doi:10.1590/1414-431X2026e15550)

Figure S1. Representative traces showing the negative chronotropic effects of the α_1-adrenoceptor antagonists alfuzosin (**A**), doxazosin (**B**), tamsulosin (**C**), and prazosin (**D**) on isolated rat right atria.

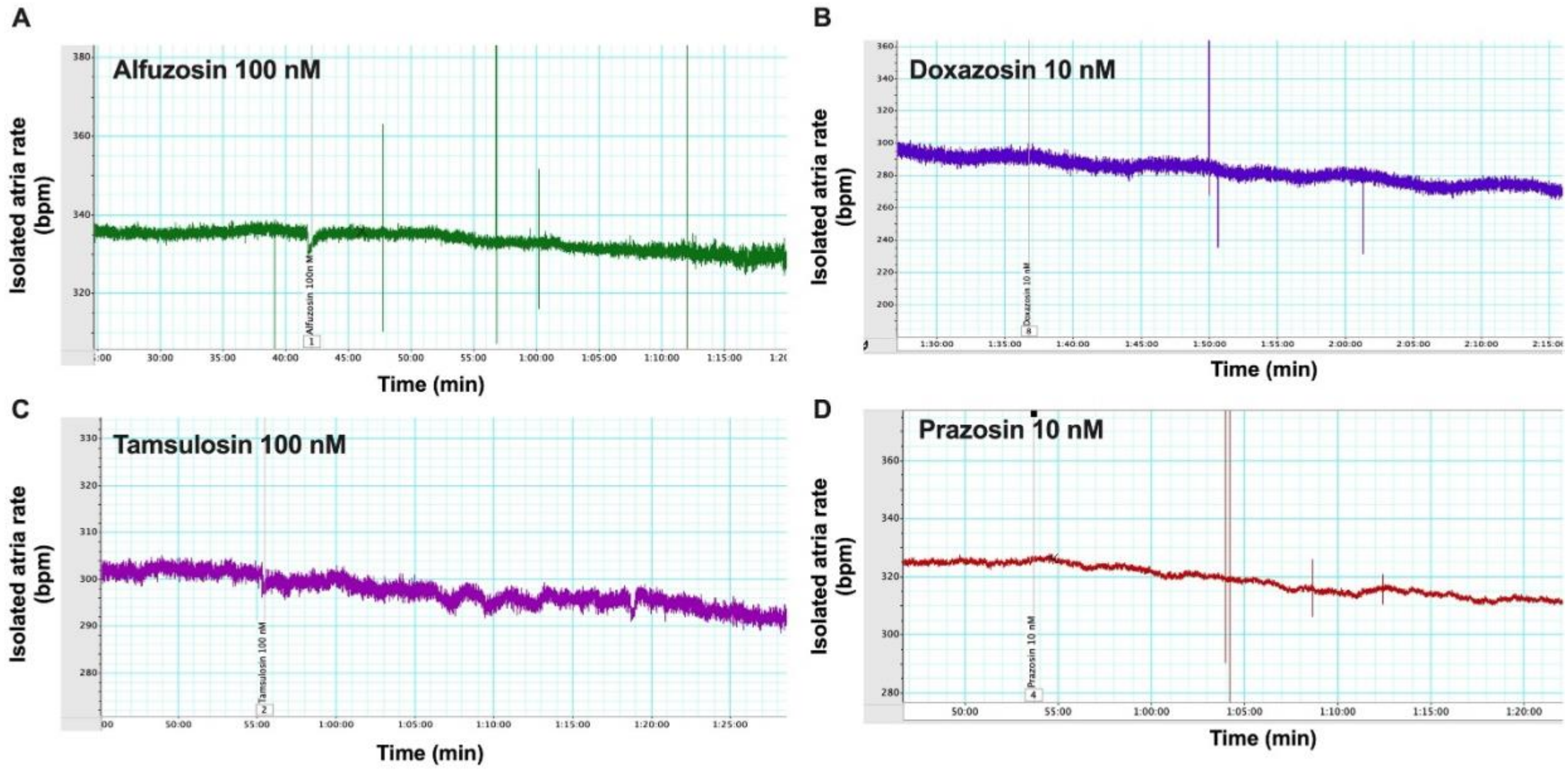

Supplement: Supplementary file 1 [file 1414-431X-bjmbr-59-e15550-suppl.pdf]
